# Association between alcohol use and inflammatory biomarkers over time among younger adults with HIV—The Russia ARCH Observational Study

**DOI:** 10.1371/journal.pone.0219710

**Published:** 2019-08-22

**Authors:** Kaku A. So-Armah, Debbie M. Cheng, Matthew S. Freiberg, Natalia Gnatienko, Gregory Patts, Yicheng Ma, Laura White, Elena Blokhina, Dmitry Lioznov, Margaret F. Doyle, Russell P. Tracy, Natalie Chichetto, Carly Bridden, Kendall Bryant, Evgeny Krupitsky, Jeffrey H. Samet

**Affiliations:** 1 Department of Medicine, Boston University School of Medicine, Boston, Massachusetts, United States of America; 2 Department of Biostatistics, Boston University School of Public Health, Boston, Massachusetts, United States of America; 3 Department of Medicine, Vanderbilt University School of Medicine, Nashville Veterans Affairs Medical Center, Tennessee Valley Healthcare System, Nashville, Tennessee, United States of America; 4 Department of Medicine, Boston Medical Center, Boston, Massachusetts, United States of America; 5 Biostatistics and Epidemiology Data Analytics Center, Boston University School of Public Health, Boston, Massachusetts, United States of America; 6 First Pavlov State Medical University, V. M. Bekhterev National Research Medical Center For Psychiatry And Neurology, St. Petersburg, Russia; 7 Department of Infectious Diseases and Epidemiology, First Pavlov State Medical University, St. Petersburg, Russia; 8 Department of Pathology and Laboratory Medicine, University of Vermont, Burlington, Vermont, United States of America; 9 National Institute on Alcohol Abuse and Alcoholism, National Institutes of Health, Bethesda, Maryland, United States of America; University of the Witwatersrand, SOUTH AFRICA

## Abstract

**Background:**

Biomarkers of monocyte activation (soluble CD14 [sCD14]), inflammation (interleukin-6 [IL-6]), and altered coagulation (D-dimer) are associated with increased mortality risk in people with HIV. The objective of the Russia Alcohol Research Collaboration on HIV/AIDS (ARCH) study was to evaluate the association between heavy alcohol use and inflammatory biomarkers over time.

**Methods:**

The study sought antiretroviral therapy naive participants with HIV (n = 350) and assessed them at baseline, 12 and 24 months. Linear mixed effects models were used to determine whether heavy drinking (self-report augmented by phosphatidylethanol [PEth], an alcohol biomarker) was longitudinally associated with IL-6, sCD14 and D-dimer adjusting for potential confounders (e.g., demographics, HIV factors, comorbid conditions).

**Results:**

Participants’ baseline characteristics were as follows: 71% male; mean age of 34 years; 87% self-reported hepatitis C; and 86% current smokers. Mean log_10_ (HIV RNA) was 4.3 copies/mL. Heavy alcohol use, based on National Institute of Alcohol Abuse and Alcoholism risky drinking criteria and PEth (versus non-heavy alcohol use) was associated with higher sCD14 (adjusted mean difference 125 ng/mL [95% CI: 42, 209]), IL-6 (ratio of means 1.35 [95% CI: 1.17, 1.55] pg/mL), and D-dimer (ratio of means 1.20 [95% CI: 1.06, 1.37] ug/mL) across the two-year follow-up.

**Conclusion:**

Among HIV+ adults, current heavy alcohol use is associated with higher sCD14, IL-6 and D-dimer over time. Since these biomarkers are associated with mortality, interventions to mitigate effects of heavy drinking on these immune processes merit consideration.

## Introduction

Alcohol use modulates immune function[1–4] contributing to increased morbidity and mortality in conditions ranging from viral hepatitis[5] to traumatic burns[6] to pulmonary infections.[7] Coupled with a disease of immune dysfunction like human immunodeficiency virus (HIV) infection,[8] the immunomodulatory effects of alcohol use are of particular concern. Biomarkers of immune processes like monocyte activation (soluble CD14 [sCD14]), inflammation (interleukin-6 [IL-6]), and altered coagulation (D-dimer), are associated with increased mortality risk among people with HIV.[9, 10] Thus factors associated with elevations in these biomarkers may present important intervention targets to reduce morbidity and mortality risk associated with HIV infection. Prior studies examining the association of heavy drinking and these biomarkers in HIV populations report inconsistent associations of alcohol use and levels of sCD14, IL-6 and D-dimer.[11–15]

Limitations of prior work include cross-sectional analyses at a single point in time that may not account for intra-person variations in these biomarkers, which are associated with multiple conditions and behaviors that co-occur with alcohol use and/or HIV infection. Using repeated measures of these biomarkers may help address this issue and may be more analytically efficient (i.e. can detect smaller differences between groups). Additional limitations of prior work include possible misclassification of alcohol use by relying solely on self-reported alcohol consumption. The current study incorporates an objective measure of alcohol use that may reduce occurrence of recall bias and/or social desirability bias, increasing the validity of alcohol assessment.[16]

The objective of the Russia ARCH (Alcohol Research Collaboration on HIV/AIDS) study was to evaluate the association between heavy alcohol use (defined based on a composite of self-report and the alcohol biomarker phosphatidylethanol (PEth)[17]) and inflammatory biomarkers over time. We hypothesized that heavy alcohol use is associated with increased sCD14, IL-6, and D-dimer.

## Methods

### Study population

Between November 2012 and October 2014, the Russia ARCH study enrolled 360 people screened as antiretroviral therapy (ART) naïve people living with HIV. Participation in the study continued irrespective of ART use post-enrollment. At screening, exposure to ART and HIV status were determined by written documentation (e.g., letter from medical provider, laboratory results, excerpts from medical histories) brought in by participants to their screening visit. Other inclusion criteria included the following: age 18–70 years; stable address within St. Petersburg or within 100km of St. Petersburg; fluency in Russian; possession of a telephone; provision of information for two contacts to assist with follow up; and ability to provide informed consent. We sought participants from a prior cohort with similar inclusion criteria,[18] as well as from clinical HIV or addiction treatment, non-clinical sites (e.g., non-governmental organizations), and via snowball recruitment in St. Petersburg. Enrollment in the current study overlapped with enrollment in a clinical trial of zinc supplementation to reduce the negative health consequences of heavy alcohol consumption (ClinicalTrials.gov identifier: NCT01934803).[19] As such, the study population was enriched for heavy drinkers at baseline. All participants provided written informed consent and Institutional Review Boards of Boston University Medical Campus and First St. Petersburg Pavlov State Medical University approved this study. Participants were followed for 24 months. They completed surveys and provided blood specimens at baseline, 12 months and 24 months. Trained research associates administered assessments in a face-to-face interview. Participants self-administered particularly sensitive sections of the assessment (e.g. HIV stigma, sexual behaviors).

### Assessments and laboratory analyses

The main exposure of interest was heavy alcohol consumption measured as a time varying covariate. This was a composite variable assessed by both the 30 Day Timeline Follow Back for alcohol use[20] and PEth.[17] Timeline Follow Back is a validated, self-reported instrument for obtaining accurate estimates and characterization of alcohol consumption. It is a time-intensive approach that uses a calendar and memory aids to mitigate recall bias (e.g., key dates like New Year’s Day can be used as anchors when recalling daily drinking). PEth is a phospholipid that accumulates in human red blood cells when a person consumes ethanol. Thus, PEth augments self-reported alcohol consumption that can be prone to recall or social desirability biases with a biochemical metric of alcohol consumption that is more objective. PEth was assayed at the United States Drug Testing Laboratories using liquid chromatography paired with tandem mass spectrometry from dried blood spots. We measured the most common PEth homologue, PEth 16:0/18:1. The limit of quantification was 8 ng/mL. PEth testing was done in two steps. The first step was for detection and the second for quantitation. Only detectable samples were retested for quantitation. We used these two methods to define alcohol use because we believe they provide the most granular self-response and biological data on quantity and frequency of recent alcohol use, the exposure we are interested in investigating.

We ascertained comorbid conditions with the following instruments: Fagerström Test for Nicotine Dependence [21], survey of co-morbidities adapted from the Veterans Aging Cohort Study patient questionnaire [22] and the HIV Risk Behavior Scale (RBS) assessing substance use and sex behaviors.[23] The Fagerström Test of Nicotine Dependence was modified to enable identification of regular smokers (i.e., participants who select “Regular smoker (smokes at least one cigarette per day (i.e., 7 cigarettes per week)” in response to the question “How would you describe your smoking behavior?” The Veterans Aging Cohort Study questionnaire enables identification of people with conditions/diseases that may confound the associations of alcohol use and biomarkers we investigating. Questions are of the form, “Has your doctor ever told you that you have any of the following: hepatitis C, diabetes or high blood sugar or "sugar"; high cholesterol, lipids, or triglycerides; kidney failure (or bad kidneys). The drug use section of the HIV Risk Behavior Scale was modified to assess use of substances in a participant’s lifetime and in the past 30-days. The questions “Have you ever used (injected, snorted, smoked, or pills)…” and “Did you use in the last 30 days…” enabled us to categorize exposure to heroin, other opioids, ephedrine, (meth)amphetamine, cocaine/crack, heroin with stimulants, cannabis, club drugs, or sedatives.

sCD14 was measured in duplicate using an ELISA (Quantikine sCD14 Immunoassay, R&D Systems Inc) with a detectable range of 40–3200 ng/mL, using a standard 200-fold sample dilution. Three controls were used, with inter-assay coefficients of variation (CVs) ranging from 8.21 to 11.45%. IL-6 was measured in duplicate using the MSD Human IL6 Ultra-sensitive Single-Plex kit (MesoScale Diagnostics, Rockville MD) which employs a sandwich ELISA technique and an electrochemiluminescent detection method, with a working range of 0.091–1498 pg/ml. Three controls, with inter-assay CVs ranging from 2.30 to 4.83%, were used. Duplicate CV’s greater than 15% were not reported. D-dimer was measured singly using the STAR automated coagulation analyzer (Diagnostica Stago), according to manufacturer’s instructions using an immuno-turbidometric assay (Liatest D-DI; Diagnostica Stago, Parsippany, NJ). Three controls with values from very low to high were analyzed, with inter-assay CVs ranging from 2.29% (high) to 18.80% (very low), were used.

### Independent variables, dependent variables and covariates

The primary exposure, heavy alcohol use, was a composite of self-reported and biochemical heavy alcohol use. We categorized participants as heavy drinkers if they either met the National Institute on Alcohol Abuse and Alcoholism (NIAAA) criteria for risky drinking *or* if they had PEth above a threshold consistent with heavy drinking.[24]

The NIAAA criteria defines heavy drinking as consuming >4 standard drinks in a day (or > 14 standard drinks/week) for men and > 3/day (or > 7/week) for women.[25] We selected a PEth threshold of ≥ 80 ng/mL because prior work suggests that this threshold corresponds to an average of at least four drinks per day with 91% sensitivity and 77% specificity.[24]

The three primary dependent variables were sCD14, IL-6 and D-dimer. IL-6 and D-dimer were natural log transformed due to skewed distributions and results were back-transformed for ease of interpretation.

Covariates included age, sex, time since HIV diagnosis, HIV RNA, regular smoking, hepatitis C, illicit drug use, study time-point, and zinc supplementation (Russia ARCH included participants from a randomized clinical trial of zinc supplementation). HIV RNA was quantified using venous blood EDTA plasma using a polymerase chain reaction (PCR)-based diagnostic with a lower limit of quantitation (LLOQ) at 500 copies/mL (AmpliSens HIVMonitor-FRT, Amplisens, Moscow, Russia). Smoking was self-reported with regular smokers reporting one cigarette per day or an average of at least seven cigarettes per week. Hepatitis C was determined by self-reported response to the question, “Has your doctor ever told you that you have hepatitis C?” Illicit drug use was determined by current (i.e., past 30 days) self-reported marijuana, cocaine, heroin, or injection drug use.

### Statistical analysis

We characterized participants by heavy drinking status across the three study time points (baseline, 12 months and 24 months). Spearman correlations between independent variables and covariates were calculated and no pair of variables included in the regression models had correlation higher than 0.40.

We applied linear mixed effects models with participant specific random intercepts and slopes to determine whether heavy drinking was associated with sCD14, IL-6 or D-dimer over the two-year follow-up. The mixed effects models incorporate multiple observations from each participant and are in general more efficient than analyses using a single time point. In addition, the models allow the incorporation of participants with partial follow-up information, thus using all available data. Additionally, the mixed effects models account for missing data conditionally on the included covariates, which are assumed to be potential predictors of the missing data. Within strata of these covariates, it is assumed that data are missing at random. It has been shown that mixed effects models and multiple imputation, a method that relies on the same assumption that the data are missing at random, produce results that are nearly indistinguishable at samples sizes of our magnitude.[26]

Differences in means are presented as the measure of association between heavy drinking and sCD14. Ratios of means are presented for the outcomes IL-6 and D-dimer as these variables were natural log transformed due to skewed distributions and regression results were then back-transformed.

The primary analyses controlled for age, sex, duration since HIV diagnosis, smoking, hepatitis C, illicit drug use, study time-point, zinc supplementation and HIV RNA. A confirmatory analysis was conducted replacing HIV RNA with ART use. Sensitivity analyses modeled alcohol as a 3-category variable (abstainers; moderate drinkers; heavy drinkers). Secondary analyses were conducted using generalized additive models[27, 28] to explore modeling alcohol use as a continuous variable. Self-reported alcohol and PEth were examined separately in these analyses. Based on the generalized additive models, linear or piecewise linear mixed effects models were then fit to evaluate whether alcohol consumption (self-reported or PEth) was associated with IL-6, sCD14 or D-dimer. For example, in the analyses of the association between self-reported alcohol consumption and IL-6, the generalized additive model suggested a non-linear relationship with a change in slope at approximately 25 drinks/week. We therefore subsequently fit a piecewise linear mixed effects model with separate slopes for alcohol use in the range of ≤ 25 drinks/week in the prior month versus alcohol use > 25 drinks/week in the prior month and tested each slope separately. Two-tailed tests and a significance level of 0.05 were used for all hypothesis testing. All analyses were conducted using SAS 9.3.

## Results

Of 360 participants enrolled at baseline, nine were later found to be HIV uninfected,[29] one was unable to provide blood specimens and one did not have complete data at baseline. Of 349 participants who had complete data available at baseline, 230 had complete data at 12 months, and 226 had complete data at 24 months; 48 died prior to completing the study. Participants’ baseline characteristics were as follows (Table 1): 71% male; mean age of 34 years; 87% self-reported hepatitis C; 86% current smokers. Mean log_10_(HIV RNA) was 4.3 copies/mL. Self-reported inflammatory conditions of aging like diabetes and kidney failure were uncommon. Recent illicit drug use was more common among heavy drinkers compared to non-heavy drinkers. Thirteen (4%) enrolled participants were later found to have had prior ART exposure at baseline; they were not excluded. By 12 months, 21% of participants were on ART; by 24 months, 35% of participants were on ART. Compared to participants with complete data, those with missing data had a higher prevalence of illicit drug use, higher PEth, greater alcohol consumption and higher sCD14, IL-6 and D-dimer at baseline (S1 Table).

**Table 1:** Characteristics of study population of people with HIV at baseline, 12 months and 24 months.

	Baseline		12 months		24 months	
	Non-Heavy drinkers (N = 79)	Heavy drinkers (N = 270)	Non-Heavy drinkers (N = 91)	Heavy drinkers (N = 139)	Non-Heavy drinkers (N = 102)	Heavy drinkers (N = 124)
**Demographics**						
Mean age (SD), years	33.9 (5.5)	33.7 (5.6)	35.2 (6.9)	34.5 (5.2)	36.5 (6.4)	35.1 (5.4)
Female	22 (27.8%)	79 (29.3%)	30 (33.0%)	42 (30.2%)	28 (27.5%)	49 (39.5%)
**HIV-related**						
Years since HIV diagnosis	7.5 (4.2)	7.0 (4.9)	7.6 (4.5)	7.7 (4.6)	8.9 (4.8)	8.8 (4.6)
Antiretroviral therapy exposure (past 6 months)	1 (1.3%)	12 (4.4%)	28 (30.8%)	20 (14.4%)	49 (48.0%)	30 (24.2%)
HIV viremia (log10) copies/mL	4.0 (1.1)	4.3 (1.1)	3.8 (1.3)	4.2 (1.2)	2.7 (1.4)	3.7 (1.4)
**Other inflammatory conditions**						
Regular smoker	62 (78.5%)	237 (87.8%)	63 (69.2%)	125 (89.9%)	71 (69.6%)	113 (91.1%)
Illicit drug use (past 30 days)	25 (31.6%)	119 (44.1%)	11 (12.1%)	63 (45.3%)	20 (19.6%)	57 (46.0%)
Has your doctor ever told you that you have hepatitis C?	73 (92.4%)	230 (85.2%)	79 (86.8%)	122 (87.8%)	83 (81.4%)	114 (91.9%)
Diabetes or high blood sugar or "sugar"	3 (3.8%)	1 (0.4%)	0 (0.0%)	3 (2.2%)	1 (1.0%)	2 (1.6%)
High cholesterol, lipids, or triglycerides	4 (5.1%)	7 (2.6%)	5 (5.5%)	4 (2.9%)	5 (4.9%)	3 (2.4%)
Kidney Failure (or bad kidneys)	1 (1.3%)	8 (3.0%)	0 (0.0%)	2 (1.4%)	5 (4.9%)	6 (4.8%)
Mean BMI, SD kg/m^2^	22.9 (3.3)	22.9 (3.1)	22.9 (3.1)	22.6 (3.7)	23.4 (3.7)	23.0 (3.5)
**Alcohol use**						
Median [p25, p75] PEth (continuous) (ng/mL)	1.0 [1.0, 22.0]	98.5 [22.0, 256.0]	1.0 [1.0, 17.0]	179.0 [75.0, 369.0]	1.0 [1.0, 17.0]	229.0 [112.0, 493.0]
Median [p25, p75] number of drinks per week (past 30 days)	0.0 [0.0, 1.3]	15.7 [9.3, 28.4]	0.0 [0.0, 0.0]	8.5 [3.9, 26.5]	0.0 [0.0, 0.4]	10.4 [2.7, 28.7]
Median [p25, p75] number of drinking days (past 30 days)	0.0 [0.0, 3.0]	13.0 [10.0, 19.0]	0.0 [0.0, 0.0]	9.0 [4.0, 18.0]	0.0 [0.0, 1.0]	8.0 [2.5, 17.0]
Median [p25, p75] number of heavy drinking days (past 30 days)	0.0 [0.0, 0.0]	5.0 [1.0, 13.0]	0.0 [0.0, 0.0]	3.0 [0.0, 12.0]	0.0 [0.0, 0.0]	2.0 [1.0, 12.0]
Median [p25, p75] number of days since last drink (past 30 days)^a^	30.0 [2.0, 30.0]	1.0 [0.0, 2.0]	30.0 [30.0, 30.0]	1.0 [0.0, 3.0]	30.0 [22.0, 30.0]	1.0 [0.0, 5.5]

^a^ Interpret values as participants having at least this number of days since last drink within the prior 30 days.

Heavy drinking was common (77%) at baseline. Median number of drinks per week in the prior 30 days was 11.5 at baseline; 2.9 at 12 months; and 2 at 24 months (Table 1) reflecting the enrichment for heavy drinkers at baseline in the zinc supplementation trial.

PEth testing revealed 20 instances wherein participants self-reported no heavy drinking at baseline but had biochemical evidence suggesting they were drinking heavily. In these instances, participants were categorized as heavy drinkers.

**Fig 1:**
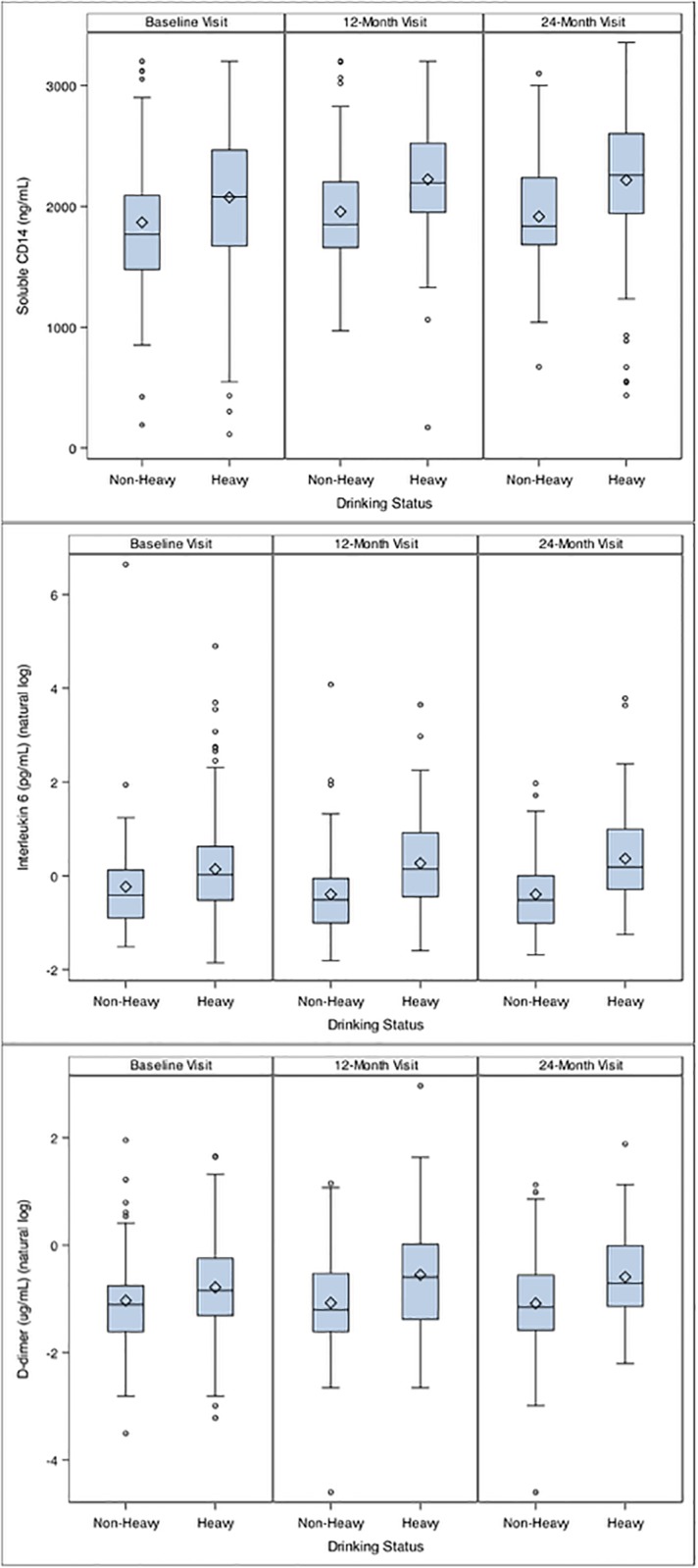
Distribution of soluble CD14 (above) interleukin-6 (middle) and D-dimer (below) by heavy drinking status at baseline 12 and 24 months.

**Fig 2:**
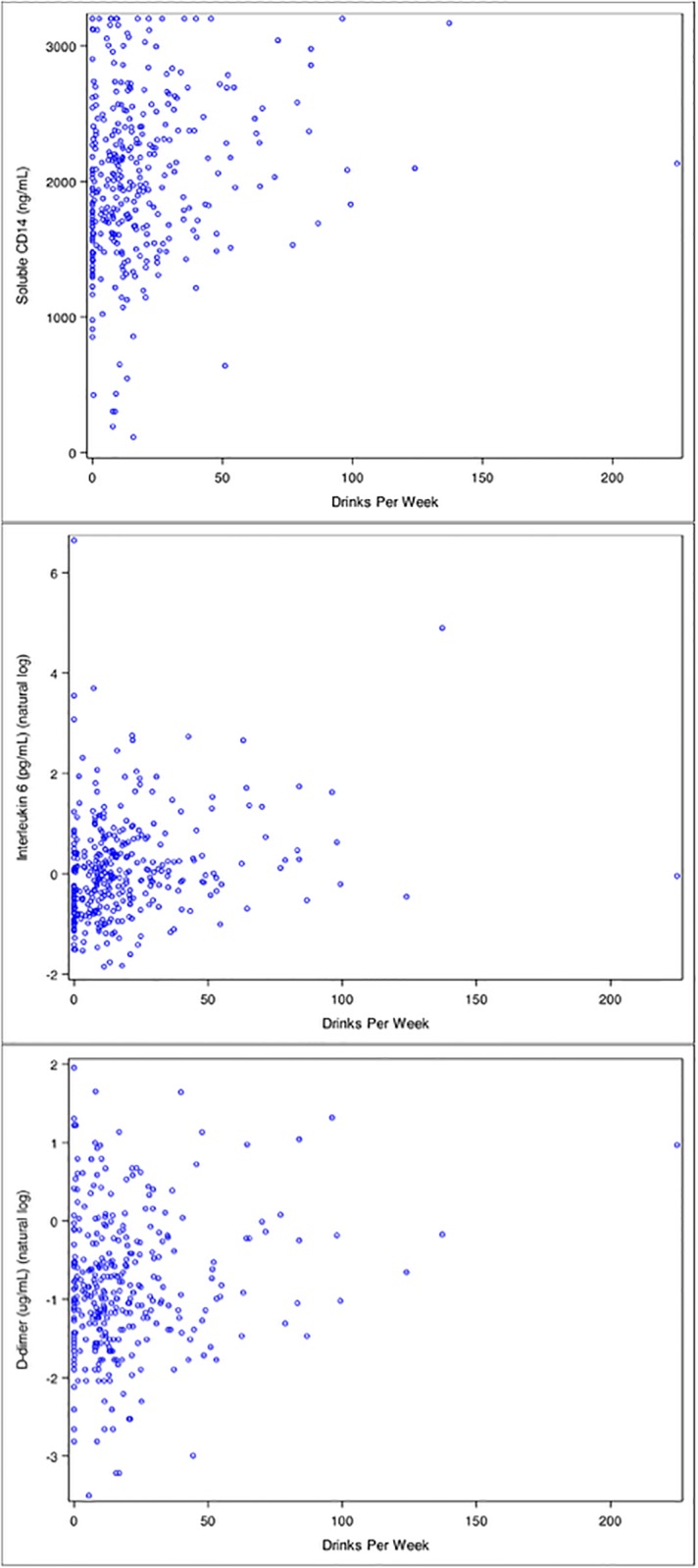
Distribution of soluble CD14 (above), interleukin-6 (middle) and D-dimer (below) by drinks per week.

Unadjusted levels of sCD14, IL-6 and D-dimer were higher among heavy drinkers compared to non-heavy drinkers (p<0.05) at baseline (Figs 1 and 2).

In adjusted mixed effects regression analyses, sCD14 was significantly higher for heavy drinkers compared to non-heavy drinkers over time (adjusted mean difference 125 ng/mL [95% CI: 42, 209], p-value<0.01; Table 2). The same was true for IL-6 (ratio of means 1.35 [95% CI: 1.17, 1.55] pg/mL, p-value<0.01; Table 3) and D-dimer (ratio of means 1.20 [95% CI: 1.06, 1.37] ug/mL, p-value<0.01; Table 4). The ratio of means is interpreted as follows using IL-6 as an example: mean IL-6 for heavy drinkers was 35% higher than that for non-heavy drinkers. In sensitivity analyses, heavy drinking remained associated with elevated sCD14, IL-6 and D-dimer when abstainers and moderate drinkers were included as separate groups (S2 Table).

**Table 2:** Results of linear mixed effects models evaluating association between current heavy drinking and sCD14 over time among ART naïve people with HIV.

	Outcome: sCD14			
	Unadjusted Mean Difference (95% CI) N = 350	p-value	Adjusted Mean Difference (95% CI) N = 349	p-value
Heavy drinking	173.7 (91.0, 256.4)	<0.001	125.2 (41.8, 208.5)	0.003
Study timepoint (one month increase)	5.1 (1.6, 8.7)	0.005	7.5 (3.5, 11.5)	<0.001
Female sex			227.7 (130.1, 325.3)	<0.001
Age (years), per 1 year increase			9.4 (1.5, 17.2)	0.020
Years since HIV diagnosis			12.9 (3.0, 22.7)	0.011
Current regular smoker			154.2 (41.7, 266.7)	0.008
Illicit drug use (past 30 days)			100.3 (16.0, 184.5)	0.020
Hepatitis C			88.6 (-52.1, 229.3)	0.216
HIV viremia (log10) copies/ml			63.3 (33.0, 93.5)	<0.001
ZINC intervention			Estimate not reported due to ongoing clinical trial analysis	--

**Table 3:** Results of linear mixed effects models evaluating association between current heavy drinking and IL-6 over time among ART naïve people with HIV.

	Outcome: IL-6^a^			
	Unadjusted Ratio of Means (95% CI) (N = 350)	p-value	Adjusted Ratio of Means (95% CI) (N = 349)	p-value
Heavy drinking	1.49 (1.29, 1.71)	<0.001	1.35 (1.17, 1.55)	<0.001
Study timepoint (one month increase)	1.01 (1.00, 1.01)	0.023	1.01 (1.01, 1.02)	<0.001
Female sex			1.07 (0.91, 1.27)	0.421
Age (years)			1.02 (1.00, 1.03)	0.010
Years since HIV diagnosis			1.01 (0.99, 1.03)	0.179
Current regular smoker			1.52 (1.26, 1.84)	<0.001
Illicit drug use (past 30 days)			1.28 (1.11, 1.47)	<0.001
Hepatitis C			1.31 (1.03, 1.66)	0.028
HIV viremia (log10)			1.19 (1.13, 1.25)	<0.001
ZINC intervention			Estimate not reported due to ongoing clinical trial analysis	--

^a^ Results back-transformed to original units after regression analyses of log-transformed values

**Table 4:** Results of linear mixed effects models evaluating association between current heavy drinking and D-dimer over time among ART naïve people with HIV.

	Outcome: D-dimer^a^			
	Unadjusted Ratio of Means (95% CI) (N = 350)	p-value	Adjusted Ratio of Means (95% CI) (N = 349)	p-value
Heavy drinking	1.29 (1.13, 1.47)	<0.001	1.20 (1.06, 1.37)	0.006
Study timepoint (one month increase)	1.01 (1.00, 1.01)	0.019	1.01 (1.01, 1.02)	<0.001
Female sex			1.32 (1.11, 1.55)	0.001
Age (years)			1.01 (1.00, 1.03)	0.049
Years since HIV diagnosis			1.01 (0.99, 1.02)	0.550
Current regular smoker			1.22 (1.02, 1.47)	0.028
Illicit drug use (past 30 days)			1.07 (0.94, 1.23)	0.302
Hepatitis C			1.29 (1.02, 1.64)	0.031
HIV viremia (log10)			1.14 (1.09, 1.19)	<0.001
ZINC intervention			Estimate not reported due to ongoing clinical trial analysis	--

^a^ Results back-transformed to original units after regression analyses of log-transformed value

**Fig 3:**
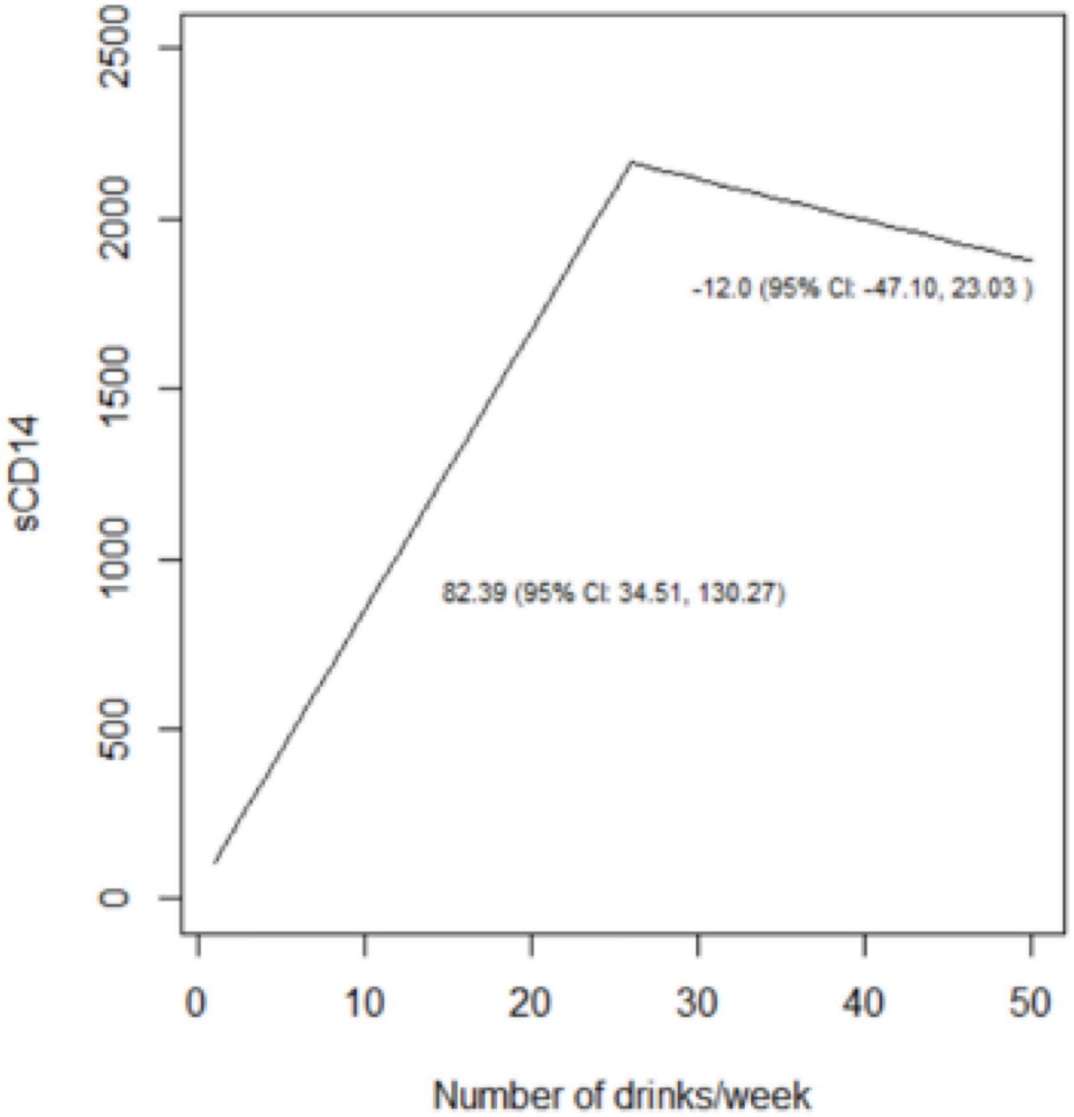
Predicted values from piecewise linear mixed effects models evaluating the association between self-reported drinks per week and sCD14. Separate slopes (95% confidence intervals) are reported for alcohol consumption of less than or equal to 25 drinks/week in the past month vs. consumption of greater than 25 drinks/week in the past month.

**Fig 4:**
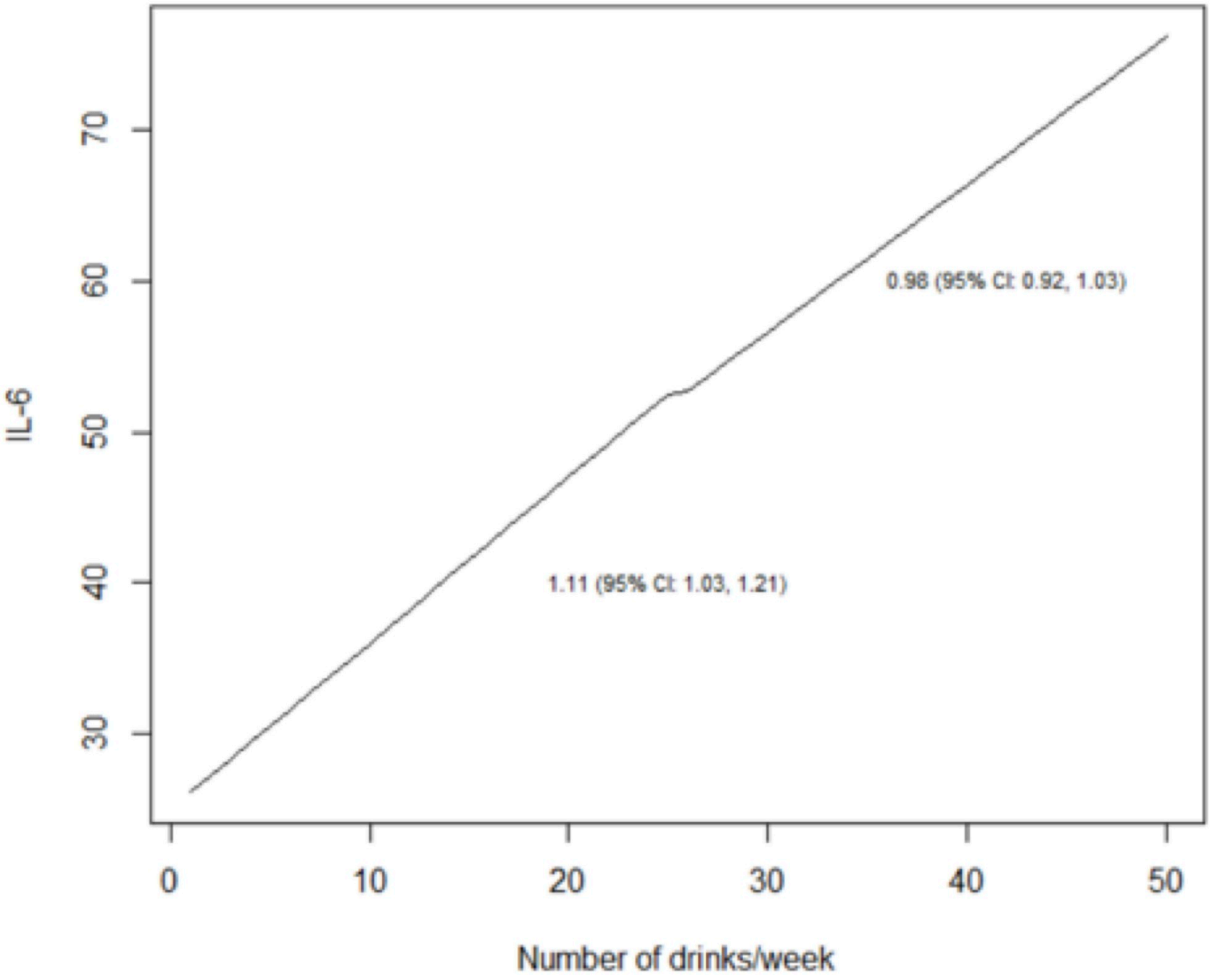
Predicted values from piecewise linear mixed effects models evaluating the association between self-reported drinks per week and IL-6. Separate slopes (95% confidence intervals) are reported for alcohol consumption of less than or equal to 25 drinks/week.

**Fig 5:**
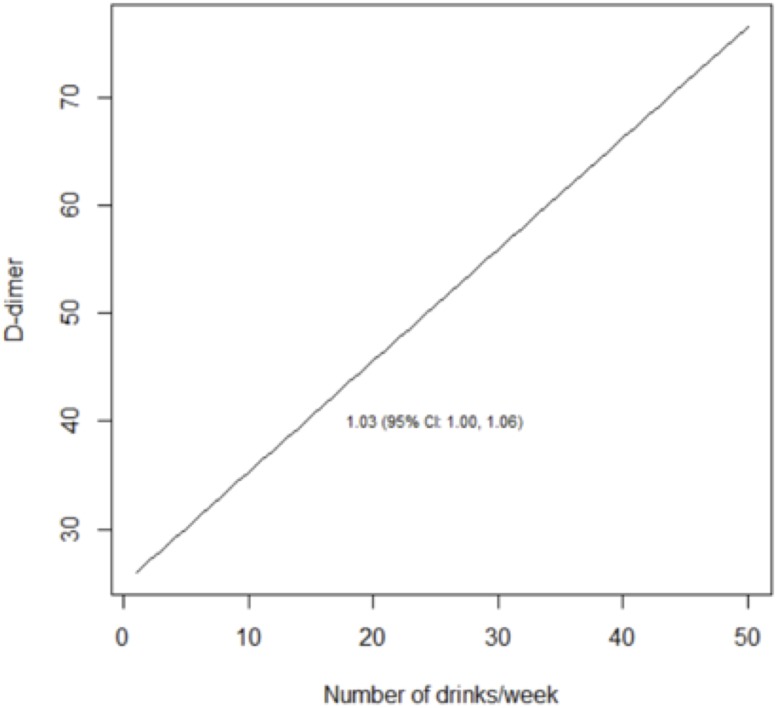
Predicted values from linear mixed effects models evaluating the association between self-reported drinks per week and D-dimer.

**Fig 6:**
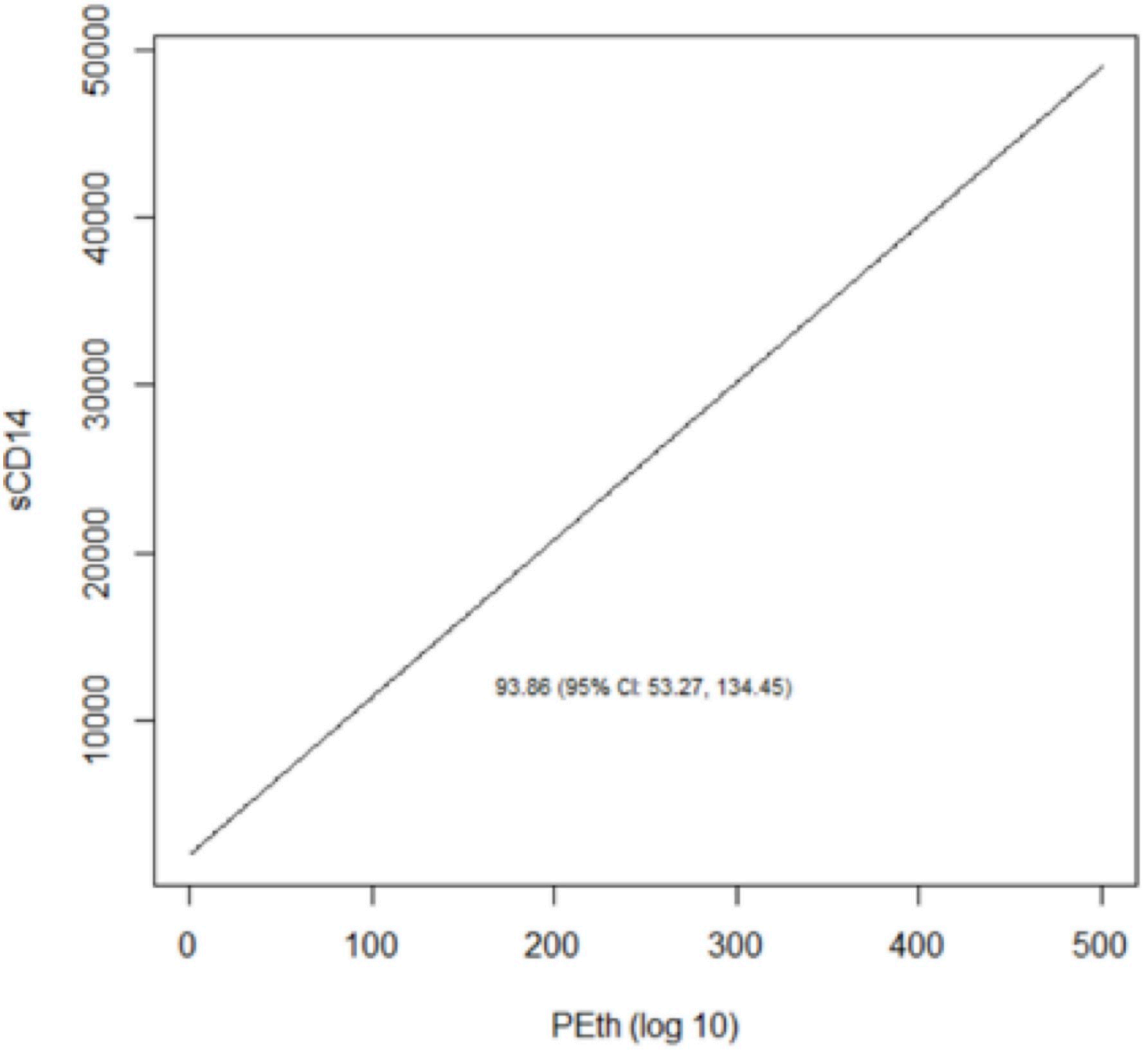
Predicted values from linear mixed effects models evaluating the association between PEth and sCD14.

**Fig 7:**
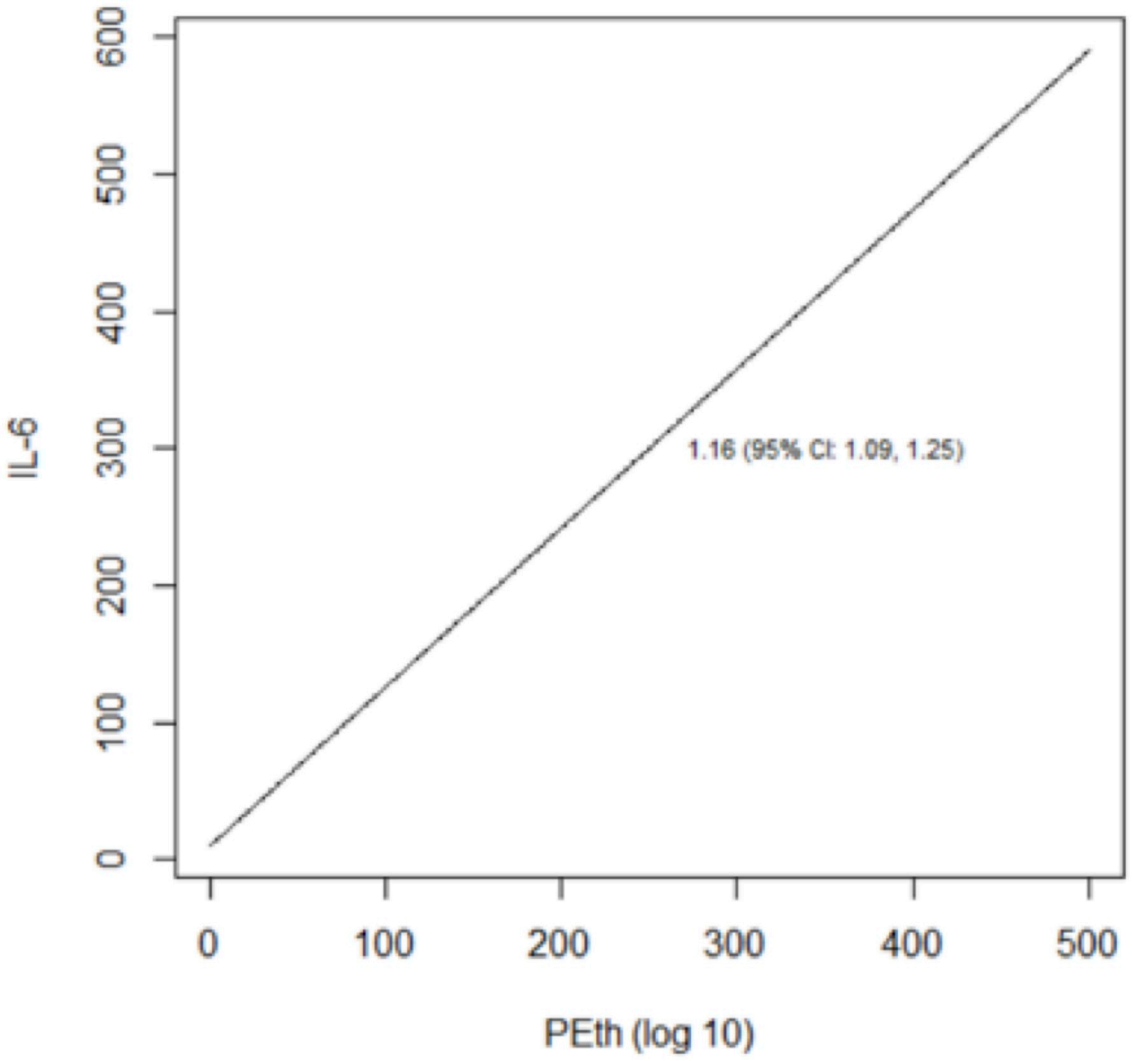
Predicted values from linear mixed effects models evaluating the association between PEth and IL-6.

**Fig 8:**
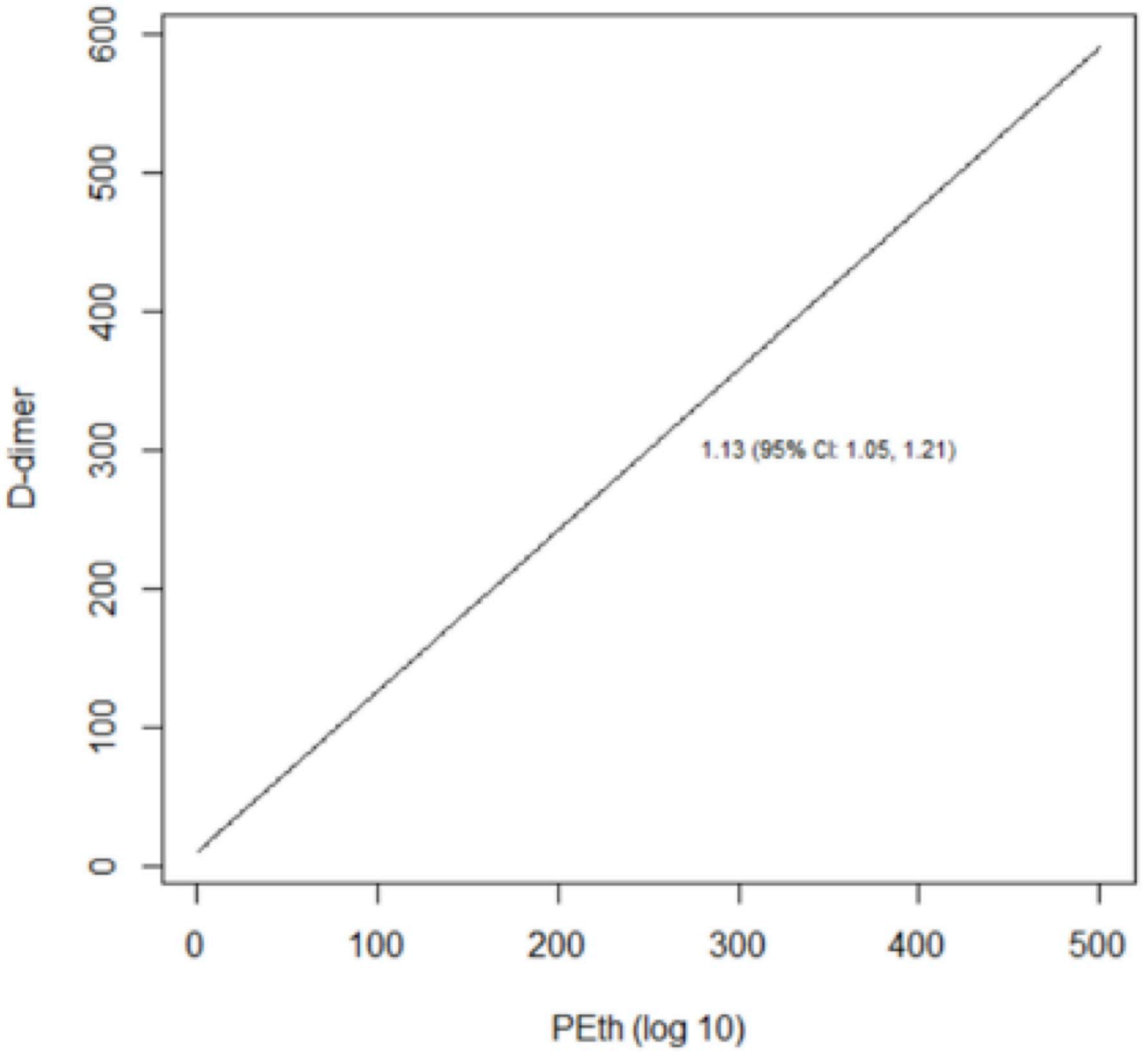
Predicted values from linear mixed effects models evaluating the association between PEth and D-dimer.

We reached similar conclusions when we analyzed alcohol use as a continuous variable (Table 5; Figs 3–8). For self-reported drinking, the relationship between alcohol consumption and both IL-6 and sCD14 appeared non-linear and therefore piecewise linear mixed effects models were fit to the data. For both IL-6 and sCD14, a separate slope was fit for alcohol consumption of less than or equal to 25 drinks/week in the past month vs. consumption of greater than 25 drinks/week in the past month. Alcohol consumption of ≤25 drinks/week occurred in 674 observations while consumption >25 drinks/week occurred for 151 observations. For alcohol consumption in the range of ≤ 25 drinks per week, there was a significant association with IL-6. For those who drank more than 25 drinks/week, we did not detect a significant association. For sCD14, there was also a significant linear relationship with alcohol consumption in the range of ≤ 25 drinks/week in the past month. For those who drank more than 25 drinks per week, we did not detect a significant association with sCD14. The association between number of drinks per week (in the prior 30 days) and log(D-dimer) appeared linear across the range of number of drinks per week although it did not reach statistical significance.

**Table 5:** Results of adjusted linear and piecewise linear mixed effects models evaluating association between a) number of drinks/week (prior 30 days) b) PEth (log10 transformed) and sCD14, IL-6 and D-dimer over time.

Independent variable	sCD14		IL-6		D-dimer	
	Adjusted Mean Difference (95% CI)	P-value	Adjusted Ratio of Means (95% CI)	P-value	Adjusted Ratio of Means (95% CI)	P-value
a) Number of drinks/week (past month)	82.39 (34.51, 130.27) ^a^	0.0009	1.11 (1.03, 1.21) ^a^	0.006	1.03 (1.00, 1.06) ^c^	0.08
	-12.03 (-47.10, 23.03) ^b^	0.50	0.98 (0.92, 1.03) ^b^	0.41		
b) PEth (log10)	93.86 (53.27, 134.45) ^c^	<0.0001	1.16 (1.09, 1.25) ^c^	<0.0001	1.13 (1.05, 1.21) ^c^	0.0006

^a^ Piecewise linear mixed effects model with alcohol consumption < = 25 drinks/week in past month

^b^ Piecewise linear mixed effects model with alcohol consumption >25 drinks/week in past month

^c^ Linear mixed effects model

Results reported per 10 unit increase in self-reported drinking and per 1 unit increase in log(10) of PEth.

The relationship between log_10_ PEth level and each of the three outcomes appeared linear and was statistically significant in all cases.

## Discussion

Among younger people with HIV in Russia, heavy alcohol use was independently associated with increased biomarkers of monocyte activation (sCD14), systemic inflammation (IL-6), and altered coagulation (D-dimer) over time.

The current study makes three important contributions to our understanding of adverse health consequences of heavy drinking among people with HIV. First, it shows that even in the presence of ongoing HIV viral replication, >85% smoking and hepatitis C prevalence, and 41% illicit drug use prevalence, heavy drinking is independently associated with biomarkers of monocyte activation, inflammation and altered coagulation across the two-year follow-up, during which some participants started antiretroviral therapy and experienced reductions in HIV viremia. Additionally, we observed potentially non-linear relationships in the association between self-reported alcohol use measured as a continuous variable and sCD14 and IL-6. Whether this finding is indicative of a real biological threshold is unclear. Given that the analogous associations for PEth appeared linear, another possible explanation for our findings is bias in self-reported alcohol use. Further support for this assertion is from our finding that the association of alcohol use as a continuous variable and D-dimer only reached statistical significance for the PEth analysis and not for the self-reported alcohol use analysis.

Published literature assessing longitudinal measurements of sCD14, D-dimer or IL-6 among people with HIV who drink is limited. One pilot study, among 21 ART-experienced, virologically suppressed men with HIV, measured alcohol use and sCD14 three months apart.[11] Heavy drinking was defined as in the present study but no biochemical measurement of alcohol use was obtained. Participants in the pilot study were selected from a larger alcohol intervention trial if they maintained heavy drinking or reduced alcohol consumption by ≥50%. In repeated-measures cross-sectional analyses similar to our analytic approach, alcohol consumption was positively associated with increased sCD14 levels. These results are consistent with our findings in Russia ARCH despite important differences between the two studies in the use of PEth to define heavy drinking, and exposure to ART and HIV viremia.

Prior cross-sectional studies have reported mixed results on the association between alcohol consumption and sCD14, IL-6 and D-dimer.[11–15] Compared to abstinent Ugandans in the Uganda ARCH cohort, unhealthy drinkers [AUDIT-C≥3 (women) ≥4 (men) or PEth>50ng/mL] had higher sCD14 but similar IL-6 and D-dimer.[12] In the SUN Study, heavy episodic alcohol use (≥5 drinks on one occasion in prior 30 days) was associated with lower D-dimer but was not associated with sCD14.[13] In a cohort of older veterans with and without HIV, we have previously reported significant associations of current hazardous drinking and past drinking (versus infrequent/moderate drinking) with IL-6 but not sCD14 or D-dimer.[30] The discrepant findings may reflect differences in alcohol use measurement, ART status, geographical environmental exposures, or referent groups (e.g., abstainers vs. non-heavy drinkers).

Second, we observed these findings in a relatively young HIV population with low prevalence of chronic, pro-inflammatory diseases of aging (e.g., diabetes, obesity, renal disease).[30] The low prevalence of these diseases of aging reduces potential confounding in assessing how heavy drinking impacts biomarkers of monocyte activation, inflammation and altered coagulation. This increases confidence in the accuracy of effects we estimate in the current study.

Third, the most recent and strongest evidence to date suggests that heavy drinking does not accelerate CD4+ T-cell decline [31]; yet the current study shows important adverse relationships between heavy drinking and immune parameters. These findings may not be conflicting as although CD4+ T-cell count may not decline with heavy drinking, other markers of inflammation are increased, raising the possibility that heavy alcohol use conveys adverse consequences for people with HIV.

This study has limitations that warrant discussion. First, monocyte activation, inflammation and altered coagulation are complex immune processes that cannot be fully captured by single biomarkers. Second, we did not have biomarkers reflecting anti-inflammatory or immunoregulatory processes (e.g., interleukin 10, transforming growth factor beta) for more comprehensive insight into the effects of alcohol consumption on inflammation in HIV. Third, our PEth thresholds consistent with four drinks per day had 77% specificity in prior studies [24] and did not consider sex specific heavy drinking criteria. Fourth, we did not have complete data on all 350 participants at baseline, 12 and 24 months. This is a potential source of bias since those with missing data had important differences from those without missing data (e.g., those with missing data had a higher prevalence of illicit drug use, higher PEth, greater alcohol consumption and higher sCD14, IL-6 and D-dimer at baseline). Importantly, death explained the missingness in data for about 40% of those missing data after baseline. Finally, as with all observational studies, we cannot exclude the possibility of residual confounding. The strength of the research is based on the rigorous measurement of alcohol exposure by the combined used of the 30 Day Timeline Follow Back for alcohol use[20] and PEth.[17]. This provided a measure of alcohol exposure that may have mitigated some of the bias from self-reporting alcohol use alone.

In conclusion, heavy alcohol use was independently associated with higher IL-6, sCD14 and D-dimer over time in a cohort of relatively young Russians living with HIV. Since sCD14, IL-6 and D-dimer predict morbidity and mortality among people with HIV, interventions to mitigate effects of heavy drinking on these immune processes merit consideration. Additionally, examining the consequences of decreasing or increasing alcohol use on these and other inflammatory biomarkers is warranted.

## Supporting information

S1 Table Comparison of baseline characteristics by data completeness. (DOCX)

S2 Table Results of adjusted linear mixed effects models evaluating association between current heavy drinking (vs moderate drinking and abstinence as separate categories) and sCD14. IL-6 and D-dimer over time among ART naïve people with HIV.
(DOCX)

S1 Dataset Analysis dataset from the Russian ARCH cohort. (CSV)
