# Supplementary material for: Association between alcohol use and inflammatory biomarkers over time among younger adults with HIV—The Russia ARCH Observational Study
Source: PLoS One. 2019 Aug 22;14(8):e0219710. doi: 10.1371/journal.pone.0219710 (PMC6705834; doi:10.1371/journal.pone.0219710)
Supplement: S1 Table — (DOCX) [file pone.0219710.s001.docx]

*Appendix Table 1: Comparison of baseline characteristics by data completeness*

|  | **Overall** | **Complete data availability** | | | **p-value** |
| --- | --- | --- | --- | --- | --- |
|  |  | **Baseline only** | **Baseline & 12 months only** | **Baseline, 12 & 24 months** |  |
| **Demographics** |  |  |  |  |  |
| Mean age (SD), years | 33.7 (5.6) | 33.5 (4.9) | 33.9 (5.0) | 33.7 (5.9) | 0.93 |
| Female | 102 (29.1%) | 17 (21.3%) | 8 (18.2%) | 77 (34.1%) | 0.02 |
| **HIV-related** |  |  |  |  |  |
| Years since HIV diagnosis | 7.1 (4.7) | 8.2 (4.8) | 6.4 (4.8) | 6.9 (4.7) | 0.06 |
| Antiretroviral therapy exposure (past 6 monnths) | 13 (3.7%) | 3 (3.8%) | 1 (2.3%) | 9 (4.0%) | 0.86 |
| HIV viremia (log10) copies/mL | 4.3 (1.1) | 4.4 (1.1) | 4.3 (1.0) | 4.2 (1.1) | 0.47 |
| **Other inflammatory conditions** |  |  |  |  |  |
| Regular smoker | 300 (85.7%) | 76 (95.0%) | 40 (90.9%) | 184 (81.4%) | 0.01 |
| Illicit drug use (past 30 days) | 144 (41.1%) | 41 (51.3%) | 22 (50.0%) | 81 (35.8%) | 0.02 |
| Has your doctor ever told you that you have hepatitis C? | 304 (86.9%) | 72 (90.0%) | 38 (86.4%) | 194 (85.8%) | 0.64 |
| Diabetes or high blood sugar or "sugar" | 4 (1.1%) | 0 (0.0%) | 0 (0.0%) | 4 (1.8%) | 0.33 |
| High cholesterol, lipids, or triglycerides | 11 (3.1%) | 1 (1.3%) | 1 (2.3%) | 9 (4.0%) | 0.46 |
| Kidney Failure (or bad kidneys) | 9 (2.6%) | 0 (0.0%) | 3 (6.8%) | 6 (2.7%) | 0.07 |
| BMI | 22.9 (3.1) | 23.0 (3.0) | 22.3 (2.4) | 23.0 (3.3) | 0.41 |
| **Alcohol use** |  |  |  |  |  |
| Median [p25, p75] PEth (continuous) (ng/mL) | 53.0 [1.0, 180.0] | 54.5 [5.0, 245.5] | 78.0 [28.0, 247.5] | 47.0 [1.0, 161.0] | <0.01 |
| Median [p25, p75] number of drinks per week (past 30 days) | 11.5 [3.9, 22.8] | 15.7 [7.6, 25.7] | 15.3 [7.9, 28.9] | 10.9 [1.3, 20.9] | 0.02 |
| Median [p25, p75] number of drinking days (past 30 days) | 12.0 [5.0, 17.0] | 13.0 [9.0, 19.5] | 13.0 [10.0, 21.0] | 10.0 [3.0, 15.0] | <0.01 |
| Median [p25, p75] number of heavy drinking days (past 30 days) | 2.0 [0.0, 10.0] | 3.0 [1.0, 13.0] | 2.5 [0.0, 13.5] | 2.0 [0.0, 8.0] | 0.27 |
| Median [p25, p75] number of days since last drink (past 30 days)^a^ | 1.0 [0.0, 5.0] | 0.0 [0.0, 2.0] | 1.0 [0.0, 2.5] | 2.0 [0.0, 8.0] | 0.02 |
| **Inflammatory biomarkers** |  |  |  |  |  |
| Mean [SD] soluble CD14 (ng/mL) | 2028 (608) | 2168 (625) | 2114 (577) | 1960 (599) | 0.02 |
| Mean [SD] log(10) interleukin 6 (pg/mL) | 0.1 (1.0) | 0.4 (1.3) | 0.3 (0.9) | -0.1 (0.9) | <0.01 |
| Mean [SD] log(10) D-dimer (ug/mL) | -0.8 (0.9) | -0.7 (0.8) | -0.6 (0.9) | -1.0 (0.9) | <0.01 |

^a^ Interpret values as participants having at least this number of days since last drink within the prior 30 days
