# Supplementary material for: Association between alcohol use and inflammatory biomarkers over time among younger adults with HIV—The Russia ARCH Observational Study
Source: PLoS One. 2019 Aug 22;14(8):e0219710. doi: 10.1371/journal.pone.0219710 (PMC6705834; doi:10.1371/journal.pone.0219710)
Supplement: S2 Table — IL-6 and D-dimer over time among ART naïve people with HIV. (DOCX) [file pone.0219710.s002.docx]

*Appendix Table 2: Results of adjusted linear mixed effects models evaluating association between current heavy drinking (vs moderate drinking and abstinence as separate categories) and sCD14. IL-6 and D-dimer over time among ART naïve people with HIV*

|  | sCD14 | | IL-6 | | D-dimer | |
| --- | --- | --- | --- | --- | --- | --- |
|  | **Adjusted Mean Difference (95% CI)** | **P-value** | **Adjusted Ratio of Means (95% CI)** | **P-value** | **Adjusted Ratio of Means (95% CI)** | **P-value** |
| Moderate drinking | Ref | 0.0049 | Ref | 0.0002 | Ref | 0.0220 |
| Abstinence | -99.7 (-234.8, 35.5) |  | 0.99 (0.79, 1.23) |  | 1.03 (0.83, 1.27) |  |
| Heavy drinking | 61.7 (-58.4, 181.8) |  | 1.34 (1.10, 1.63) |  | 1.22 (1.02, 1.47) |  |
